# Feasibility of a GP delivered skin cancer prevention intervention in Australia

**DOI:** 10.1186/1471-2296-15-137

**Published:** 2014-07-28

**Authors:** Kylie Vuong, Lyndal Trevena, Billie Bonevski, Bruce K Armstrong

**Affiliations:** 1 Sydney School of Public Health, University of Sydney, Sydney, New South Wales, Australia; 2 School of Medicine and Public Health, University of Newcastle, Callaghan, New South Wales, Australia; 3 Cancer Epidemiology and Services Research, Sydney School of Public Health, The University of Sydney, Sydney, New South Wales 2006, Australia

**Keywords:** Feasibility studies, Skin neoplasms, Preventive medicine, General practice, Health behaviour

## Abstract

**Background:**

Despite years of public education, sun-related behaviours are difficult to change and a recent survey showed low levels of sun protection. In this study we evaluated the feasibility and acceptability of an opportunistic skin cancer prevention intervention in general practice.

**Methods:**

We used a controlled pre-and-post intervention design. Participants (n = 100) were recruited sequentially from patients attending two general practices in Sydney, Australia, from November to December 2010. Participants in the intervention practice (n = 50) received general practitioner delivered sun protection advice after completing a skin cancer risk assessment tool, and a sun protection pamphlet, in addition to routine care, at a single attendance. The skin cancer risk assessment tool provided three levels of risk. The general practitioner (GP) reinforced the level of risk and discussed sun protection. Participants in the control practice (n = 50) received routine care. We measured feasibility by patients’ and GPs’ participation in the intervention and time taken, and acceptability by intervention participants and GPs ratings of the intervention. We measured reported sun-related knowledge, attitudes and behaviour between the two groups at 1 and 13 months.

**Results:**

The intervention was found to be feasible within existing primary care team arrangements. Participation at baseline was 81% (108/134), and repeated participation was 88% (88/100) at 1 month and 70% (70/100) at 13 months. Participants and practitioners found the intervention acceptable. At 1 month, sun-related knowledge had increased in both patient groups, with a greater increase in the intervention group (adjusted mean difference 0.48, p = 0.034). There were no differences between groups in sun-related knowledge, attitudes and behaviour at 13 months.

**Conclusions:**

A brief opportunistic skin cancer prevention intervention in general practice is feasible and acceptable. Further research in this setting with a more intensive intervention would be justified.

## Background

Skin cancer, which includes basal cell carcinoma, squamous cell carcinoma and melanoma is the most expensive cancer in Australia [1]. Prevention guidelines recommend reducing exposure to ultraviolet (UV) radiation, wearing protective clothing, wearing a hat, and using sunscreen during peak UV periods [2]. Despite more than 25 years of public education, [3,4] a national survey of 5,412 adult respondents showed low levels of sun protection [5]. Further interventions in skin cancer prevention are needed.

Primary care-relevant counselling is associated with moderate improvements in sun protection behaviours; however these improvements are of uncertain clinical relevance [6]. For adults over 24 years, there is inadequate evidence to assess the benefits of behavioural counselling on sun protection and skin cancer prevention [7]. The US Preventive Services Task Force recommends further research on the effectiveness of counselling on sun protection behaviours and the development of new ways of administering interventions to address this evidence gap [7].

Health information that is informed by a person’s unique characteristics may be effective [8]. In primary care settings, skin cancer prevention interventions have included mailed packages [9,10] and interactive computer-based education [11]. However there are few studies that have directly evaluated physician-delivered feedback interventions on patients’ sun protection. In an early study, 37% of the intervened sample began using sun protection factor 15 sunscreen following repetitive sun protection advice given by a dermatologist [12]. More recently, greater changes in sunscreen use were shown in a group who received sun protection advice from a general practitioner (GP) in a 20 minute consultation compared to a group who received the advice by means of a letter [13,14].

General practice’s focus on comprehensive whole-person care and the steady flow of skin cancer related attendances make it an ideal setting for skin cancer prevention [15,16]. We aimed to evaluate the feasibility and acceptability of a brief skin cancer prevention intervention delivered opportunistically, and measure sun-related knowledge, attitudes and behaviour.

## Methods

The study was a pre-post design with a control group. Participants were surveyed on their sun-related knowledge, attitudes and behaviours immediately before intervention, and at 1 and 13 months later.

### Participants

#### General practitioners participants

A practice-based intervention was selected to limit potential contamination; two general practices volunteered. Both practices had past research participation and fulfilled our selection criteria: willingness of the GPs to participate and a sizeable waiting room. The intervention practice, located in the Australian State of New South Wales’ Leichhardt local government area (Index of Relative Socioeconomic Disadvantage score = 1078.9, which ranks it in the highest 10% of the State for socioeconomic status of its residents), [17] employed 9 GPs, 6 full-time and 3 part-time, 6 female and 3 male. The control practice, located in the Ashfield local government area (Index of Relative Socioeconomic Disadvantage score = 1015.4, which ranks it in the highest 30% of the State), [17] employed 8 full-time GPs, 4 female and 4 male.

#### Patient participants

Individual participants were recruited from November to December 2010 in the practice waiting room. To minimise disruption to practice operations, a research assistant screened “the most recent patient to enter the waiting room” for eligibility and invited eligible patients to participate. Recruitment was stratified with a notional quota for each half-day session of 12 patients equally distributed by sex and age (less than 50 years old or greater than 50 years old) for each session. We included patients across all skin cancer risk categories [18,19]. To be eligible, a patient had to be aged over 18 years, have the capacity to give meaningful consent and be well enough to participate. Ethical approval (No. 13129) was obtained from *The University of Sydney* Human Research Ethics Committee and informed consent was obtained from all participants.

### Intervention

The intervention involved a skin cancer risk assessment tool, GPs delivering sun protection advice informed by the patient’s skin cancer risk and a *SunSmart UV Alert* pamphlet [20].

Protection motivation theory, [21] shown to be effective in promoting sun protection, [22-24] provided the theoretical framework for our study. Protection motivation theory proposes that, when individuals are confronted with a health threat (skin cancer risk), they engage in two cognitive processes: threat appraisal and coping appraisal [21]. Components of threat appraisal include severity (perceived seriousness of skin cancer) and vulnerability (perceived risk of skin cancer); components of coping appraisal include response efficacy (perceived effectiveness of sun protection behaviours) and self-efficacy (one’s ability to execute the recommended prevention); motivation to protect one’s self is the mediating variable that directs behaviours [21]. The intervention aimed to improve sun protection behaviours by strengthening the perceived threat from skin cancer together with strong coping messages.

GPs in the intervention group were briefed collectively at 1 month and individually at 1 week before recruitment began on the skin cancer risk assessment tool, the delivery of sun protection advice and the *SunSmart UV Alert* pamphlet [20]. The skin cancer risk assessment tool was adapted from existing tools [25-27]. It provided for three levels of risk based on hair colour, skin colour, sun sensitivity, the presence of moles and personal skin cancer history (Additional file 1). Patients in the intervention group completed, unassisted, the skin cancer risk assessment tool in the waiting room and showed it to their GP in the consultation immediately following recruitment. The GPs reinforced the patients’ level of risk from the skin cancer risk assessment and discussed sun protection including sunscreen use, use of protective clothing, the importance of wearing a broad-brimmed hat, use of shade and the UV index. Each intervention patient was provided with a copy of the skin cancer risk assessment tool and a *SunSmart UV Alert* pamphlet [20]. The *SunSmart UV Alert* pamphlet included information on UV radiation, the UV index, and on sun protection [20].

Patients in the control group also completed the skin cancer risk assessment; which appeared at the end of the baseline survey. The assessment did not include any risk levels or sun protection advice and the research assistant collected it with the completed baseline survey before the GP consultation. Control patients received usual GP care; i.e. the GPs were not given any specific instructions on providing skin cancer prevention advice and provided counselling at their own discretion. In a study of US family physicians, skin cancer prevention formed a small part of the preventive workload with skin cancer prevention advice offered in only 1% of patient visits [28]. There are no corresponding Australian data.

### Measures of feasibility

Feasibility studies are small scale studies conducted to test new research plans and inform the development of larger scale studies [29,30]. Feasibility studies usually address four main components of a proposed study: process, testing the steps that are key to the success of the study; resources, assessing time and budget requirements; management, assessing human and data management needs; and scientific, assessing the likely effect of the intervention [29]. Our feasibility study focused on processes and resources. We assessed rate of invited patients’ participation in the intervention and reasons for non-participation; and the time required for participants to complete the skin cancer risk assessment tool and for the GP to deliver the sun protection advice.

### Measures of acceptability

To access acceptability, intervention group patients were asked in the follow-up survey to rate the intervention materials using a four-point Likert scale. The questions about the skin cancer risk assessment tool were: “How useful did you find the skin cancer risk checklist that was given to you in the waiting room?” and “How easy was it to complete this skin cancer risk checklist?” The question on the sun protection advice was: “How useful did you find these discussions?” The questions on the *SunSmart UV Alert* pamphlet [20] were: “How useful did you find the sun protection pamphlet that was given to you by the GP?” and “Was the sun protection pamphlet easy to read?”

Intervention group GPs were individually interviewed in February 2011. The interview was structured around sun protection advice, skin cancer risk assessment tool, and *SunSmart UV Alert* pamphlet [20].

### Measures of sun protection knowledge, attitudes and behaviours

A sun habits survey was adapted from existing surveys (Additional file 2) [31,32]. Participants completed, unassisted, a baseline survey in the waiting room before seeing the GP and follow up surveys at home at 1 and 13 months later. Each survey collected the same data on sun-related knowledge, attitudes and behaviours.

Sun-related knowledge was measured with six items using a true-false format (Items 1-6, Additional file 2). Each correct answer to a knowledge question contributed one point to a composite knowledge score. Higher scores reflect higher knowledge levels. The Cronbach alpha coefficient for the composite knowledge score was 0.38.

Sun-related attitudes, as key components of protection motivation theory, were measured using a five-point Likert scale. Perceived severity (Items 8, 9 and 10, Additional file 2), vulnerability (Items 11 and 12, Additional file 2) and self-efficacy (Items 14, 15 and 16, Additional file 2) were represented by a composite score. Response-efficacy (Item 13, Additional file 2) was represented by a single-item score. Higher scores reflect more favourable sun-related attitudes. The Cronbach alpha coefficients for the composite severity, vulnerability and self-efficacy scores were 0.46, 0.87 and 0.61 respectively.

Sun exposure was measured by asking participants to indicate the number of hours they usually spent outdoors without reference to a specific time period (Item 26, Additional file 2). Sun-related behaviours, including sunscreen use, wearing a hat, wearing a long sleeve shirt, wearing sunglasses and limiting time in the sun, were measured using a five-point Likert scale and summed as a sun protection score. The Cronbach alpha coefficient for the sun protection score was 0.65.

### Statistical methods

Multiple linear regression modelling was used to assess differences between intervention and control groups in their responses to the follow-up surveys after controlling for the baseline (first survey) value of the variable being compared, age and sex. The other baseline variables were entered into the model to test whether they contributed to the predictive ability of the model. Adjusted mean values were obtained using one-way analysis of covariance. Data analyses were conducted using SPSS-19 statistical software [33].

## Results

### Characteristics of participants

**Figure 1:**
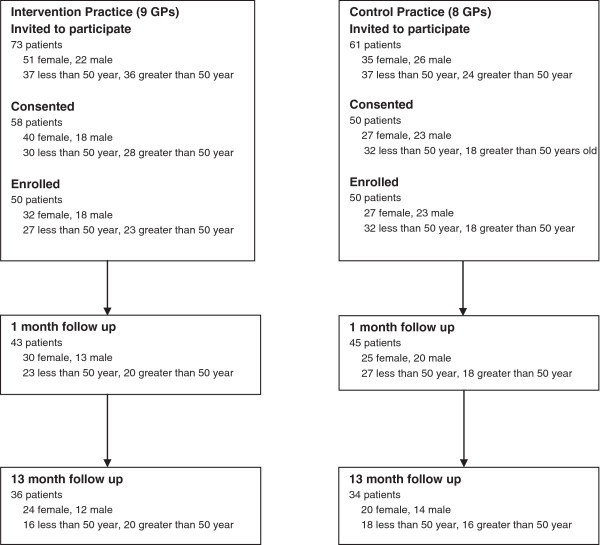
Flow diagram showing the recruitment of participants from two Sydney general practices in 2010-12.

Figure 1 shows the recruitment of participants. Among the patients who were screened and invited to participate, three were ineligible because they did not have the capacity to offer meaningful consent and one because she did not have an appointment to see the GP. Twenty two patients refused, reasons offered included being busy (12 patients) and high compliance with sun protection (2 patients). Among the 58 intervention group patients who consented to participate, 8 were called in by their GPs before they completed the baseline sun habit survey and skin cancer risk assessment tool, and therefore not enrolled.

While there were no statistically significant differences between intervention and control groups with regards to age, gender, marital status, educational level, household weekly income, country of birth, self-reported health status and skin cancer risk category, there were trends towards higher educational level, higher household income and better health status in the intervention patients than the control patients (Table 1).

**Table 1:** Descriptive information on the study sample – Sydney, Australia, 2010

	**Intervention (n = 50)**		**Control (n = 50)**		**P**^**diff**^
Variable	n	%	n	%	
Age					
<50 years	27	54	32	64	
50 years or greater	23	46	18	36	0.42
Gender					
Male	18	36	23	46	
Female	32	64	27	54	0.42
Marital status					
Married or living in a defacto relationship	33	66	30	60	
Never married/separated/ divorced/widowed	17	34	20	40	0.68
Educational level					
High school/College	9	18	16	32	
University	39	78	31	62	0.09
Household weekly income					
AUD <600	7	14	7	14	
AUD 601- 1000	8	16	11	22	
AUD 1001-2000	8	16	11	22	
Greater than AUD 2000	23	46	19	38	0.73
Country of birth					
Australia	36	72	40	80	
Other	14	28	10	20	0.48
Self-reported health status in last week					
Very poor/poor	5	10	2	4	
Fair/good	22	44	31	62	
Very good/excellent	23	46	17	34	0.16
Skin Cancer Risk Category					
Below average	4	8	2	4	
Average	9	18	9	18	
Above average	37	74	39	78	0.70

### Feasibility

Patients’ participation in the intervention was high, 81% of those invited. At 1 month follow up, the response was 86% for the intervention group and 90% for the control group. At 13 months, the response was 72% for the intervention group and 68% for the control group. All GPs in both practices participated. Six out of nine GPs from the intervention practice agreed to be interviewed upon completion of the study, the remaining three were on leave at the time of the interviews. Each intervention patient completed the skin cancer risk assessment tool unassisted in 3 to 5 minutes. The sun protection advice took 3 to 5 minutes. The intervention GPs were able to carry out the intervention within the allotted appointment times (15 minutes) and no concern was expressed about the small increase in the consultation time.

### Patient acceptability

At 1 month 95% of 43 intervention group respondents felt that the skin cancer risk assessment was useful and 98% felt it was easy to understand. Almost two thirds (71%) of the intervention group respondents remembered a discussion with their GP on skin cancer and all of them felt the discussion was useful. Similarly 97% felt the *SunSmart UV Alert* pamphlet [20] was useful and all felt it was easy to understand.

### General practitioner acceptability

All interviewed GPs (n = 6) believed they played a useful role in skin cancer prevention. All felt confident in delivering the advice and believed it was beneficial to their patients. All GPs reported the risk assessment tool as easy to understand. Four GPs reported the skin cancer risk assessment tool as useful; one GP said “It’s difficult to get GPs to do this intervention on every patient” (GP 3), and another “I’m not sure whether it’s helpful” (GP 5). All GPs reported the *SunSmart UV Alert* pamphlet [20] as useful, four reported it as easy for the patients to understand, one GP said “I’m not sure whether all the patients took them away” (GP 4), and another “There were not many chances for me to get feedback from the patient” (GP 5).

### Measures of sun-related knowledge, attitudes and behaviour

Knowledge levels were high at baseline with a mean score of 4.59 in the intervention and 4.16 in the control group out a possible total score of 6. At 1 month follow up, the intervention group showed a greater increase in knowledge score (mean difference = 0.48, p = 0.034) (Table 2). There were no material differences between groups in sun-related knowledge, attitudes or behaviour at 13 months.

**Table 2:** Sun-related knowledge, attitudes and behaviour at baseline, one and 13 month follow up - Sydney, Australia, 2010- 2012

	**Intervention**			**Control**			**Mean difference between intervention and control (95% confidence intervals)**^**b**^	**p value**
	**n**^**a**^	**Mean**	**SE**	**n**^**a**^	**Mean**	**SE**		
Sun protection behaviour								
Sun protection score^c^								
Baseline	50	16.70	0.57	50	14.9	0.49		
Follow up at 1 month	43	15.61	0.36	45	15.62	0.35	-0.004 (-1.02, 1.01)	0.99
Follow up at 13 month	37	16.64	0.35	34	16.39	0.37	0.26 (-0.78, 1.29)	0.63
Hours spent outdoors per day								
Baseline	49	2.05	0.24	49	2.66	0.28		
Follow up at 1 month	42	2.28	0.16	43	2.33	0.16	-0.06 (-0.51, 0.40)	0.81
Follow up at 13 month	37	2.07	0.22	33	2.41	0.23	-0.34 (-0.98, 0.30)	0.29
Knowledge score^d^								
Baseline	49	4.59	0.16	49	4.16	0.18		
Follow up at 1 month	41	5.03	0.16	43	4.55	0.15	0.48 ( 0.04, 0.92)	0.034
Follow up at 13 month	36	4.63	0.13	33	4.62	0.14	0.01 (-0.38, 0.40)	0.96
Sun protection attitudes^e^								
Perceived severity^f^								
Baseline	50	10.8	0.30	49	10.51	0.28		
Follow up at 1 month	43	10.85	0.21	44	10.67	0.21	0.19 (- 0.41, 0.78)	0.53
Follow up at 13 month	37	10.14	0.26	33	10.54	0.28	-0.40 (-1.16, 0.37)	0.30
Perceived vulnerability^g^								
Baseline	50	4.74	0.34	48	5.02	0.33		
Follow up at 1 month	43	4.66	0.25	43	4.27	0.25	0.40 (-0.32, 1.11)	0.28
Follow up at 13 month	37	4.89	0.37	32	5.44	0.39	-0.54 (-1.62, 0.54)	0.32
Response efficacy^h^								
Baseline	50	4.60	0.08	50	4.80	0.06		
Follow up at 1 month	43	4.34	0.10	45	4.52	0.10	-0.18 (-0.47, 0.11)	0.22
Follow up at 13 month	37	4.40	0.12	34	4.62	0.13	-0.22 (-0.58, 0.13)	0.21
Self efficacy^f^								
Baseline	50	10.22	0.30	50	9.04	0.35		
Follow up at 1 month	43	9.07	0.28	45	9.73	0.27	-0.66 (-1.45, 0.14)	0.10
Follow up at 13 month	37	7.92	0.33	34	7.68	0.35	0.25 (-0.73, 1.22)	0.62
Social norms^i^								
Baseline	50	6.22	0.21	50	5.74	0.22		
Follow up at 1 month	43	6.18	0.20	45	5.76	0.20	0.42 (-0.16, 0.99)	0.15
Follow up at 13 month	37	5.76	0.21	34	5.88	0.21	-0.12 (-0.72, 0.48)	0.69
Attractiveness of tanning^h^								
Baseline	50	3.30	0.13	50	3.38	0.12		
Follow up at 1 month	43	3.46	0.11	45	3.28	0.11	0.18 (-0.14, 0.50)	0.27
Follow up at 13 month	37	3.22	0.12	34	2.88	0.12	0.34 (-0.005, 0.69)	0.053

^a^Number of respondents with sufficient data for analysis.

^b^Adjusted for the corresponding baseline value, age and sex.

^c^Range of values was 5-25 (composite of 5 questions where 1 = never to 5 = always).

^d^Range of values was 0-6 (total number of correct answers).

^e^Expressed in terms of key components of protection motivation theory (Rogers, 1983 [21]).

^f^Range of values was 3-15 (composite of 3 questions where 1 = strongly disagree to 5 = strongly agree).

^g^Range of values was 2-10 (composite of 2 questions where 1 = less than 20% chance of skin cancer to 5 = 81-100% chance of skin cancer).

^h^Range of values was 1-5 (1 = strongly disagree to 5 = strongly agree).

^i^Range of values was 2-10 (composite of 2 questions where 1 = strongly disagree to 5 = strongly agree).

## Discussion

A brief skin cancer prevention intervention delivered opportunistically was feasible and acceptable to both intervening GPs and patients. As in previous Australian studies, [34,35] baseline knowledge levels were high. Relevant sun-related knowledge increased in both groups; and there was a greater short-term increase in relevant knowledge in the intervention group, which was not maintained. There was no evidence of a favourable change in sun-related attitudes or behaviour due to the intervention.

Our study was designed to be delivered opportunistically at a single attendance; other physician-delivered interventions have been more intensive. Robinson’s intervention involved repetitive oral and written skin cancer prevention education with clinical skin examinations at 2 weeks and 2, 6, 12 months post-surgical excision of a non-melanoma skin cancer [12]. Falk and Anderson’s intervention involved a separate 20 minute consultation with a single GP who offered oral and written skin cancer prevention education with a clinical skin examination [14]. Rat and colleagues’ melanoma prevention intervention involved a separate consultation with a single GP who offered oral and written skin cancer prevention with a clinical skin examination for patients at high risk of developing primary melanoma [36].

Improvements in sun-related behaviours are difficult to achieve. In Falk and Anderson’s study short-term differences in sunbathing and shade seeking behaviour in the group who received sun protection advice from a GP were not maintained [14]. At 3 year follow up, significant differences in sun protection behaviour were only seen for sunscreen use in the group who received sun protection advice from a GP compared to the group who received the advice by means of a letter [14]. While our observation of an early effect of the intervention on sun-related knowledge raised the possibility of behaviour change, other evidence indicates that effects of knowledge on attitudes and behaviour are not well understood, and that sun-related attitudes and behaviours are difficult to change [35]. Despite high sun-related knowledge, people often maintain their usual habits [37,38].

The main strengths of our study were the opportunistic approach, involvement of the patient’s usual GP, minimal disruption to the general practice routine and intervention delivery shortly before the Australian summer holidays. Despite the practical difficulties with follow up over summer, the response among the patients was high and all intervention GPs who were contactable agreed to be interviewed. Further, the intervention and control groups were reasonably well matched at baseline.

It was a potential limitation that six different GPs delivered the intervention and may have varied in the ways they delivered it. The study used self-reported outcomes, which are open to various biases; however previous studies show that self-reported sun protection behaviours are well correlated with readings from UV dosimeters and direct observation [39-41]. As a feasibility study, this study was insufficiently powered to observe other than a large effect. Including participants across all skin cancer risk categories may also have reduced the study’s power.

## Conclusions

An opportunistic skin cancer prevention intervention is feasible and acceptable within existing primary care arrangements. Evidence of a greater short-term increase in relevant knowledge in the intervention group was not maintained in the longer term. There was no evidence of a favourable change in sun-related attitudes or behaviour due to the intervention; although the study was insufficiently powered to observe other than large effects. The feasibility and acceptability of our approach suggests that further research in this setting, perhaps with strengthened and repeated messages, would be justified.

## Abbreviations

GP: General practitioner; UV: Ultraviolet radiation.

## Competing interests

All authors declare that they have no competing interests.

## Authors’ contributions

KV performed the statistical analysis. KV, LT, BB and BKA conceived the study and participated in its design and coordination and helped to draft the manuscript. All authors read and approved the final manuscript.

## Pre-publication history

The pre-publication history for this paper can be accessed here:

http://www.biomedcentral.com/1471-2296/15/137/prepub

## Supplementary Material

Additional file 1 Skin cancer risk assessment tool used in two Sydney general practices in 2010. Click here for file

Additional file 2 Sun habits survey. Click here for file
