# Supplementary figures and images for: Feasibility of a GP delivered skin cancer prevention intervention in Australia
Source: BMC Fam Pract. 2014 Jul 28;15:137. doi: 10.1186/1471-2296-15-137 (PMC4128422; doi:10.1186/1471-2296-15-137)

Additional file 1:Skin cancer risk assessment tool used in two Sydney general practices in 2010


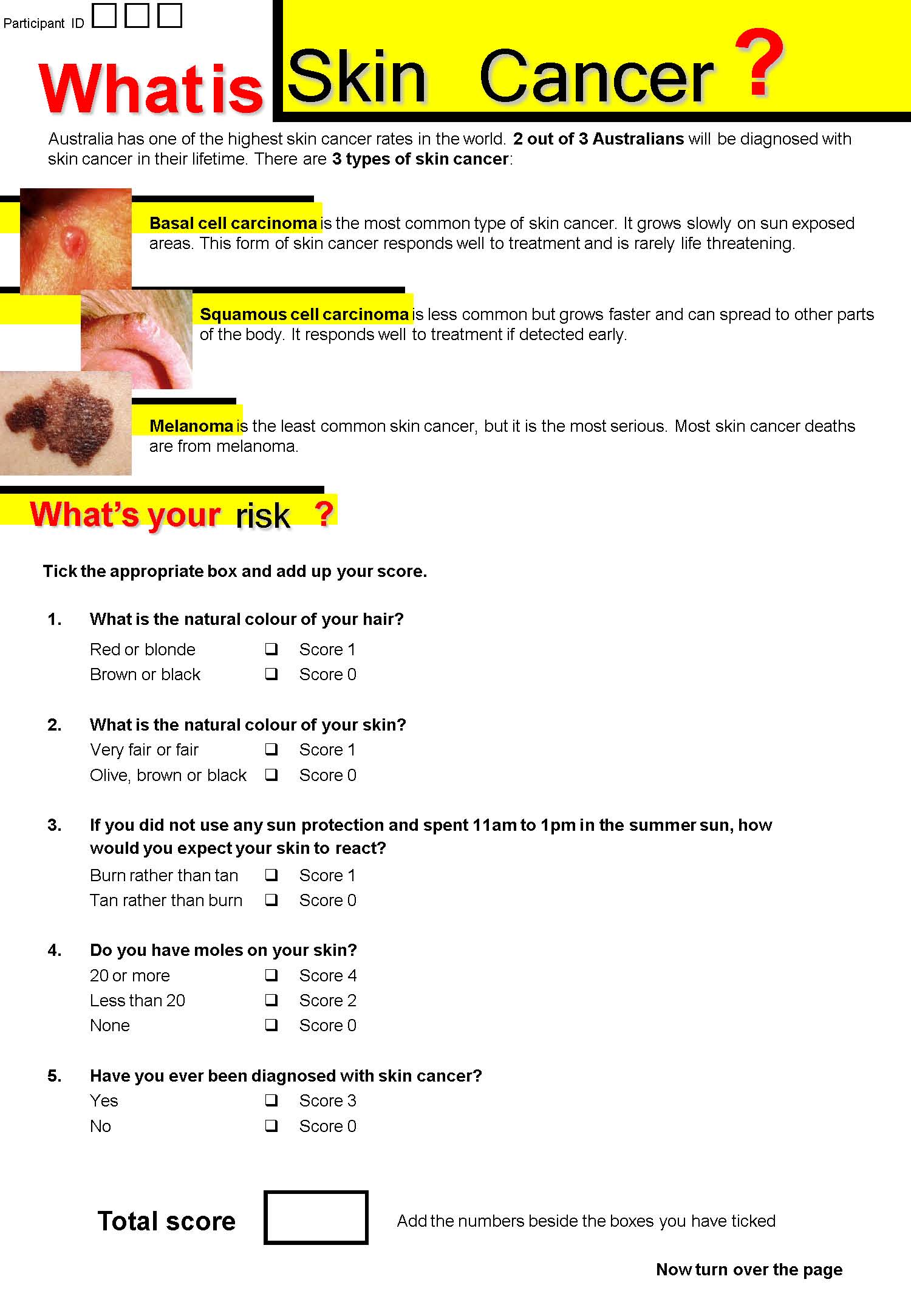


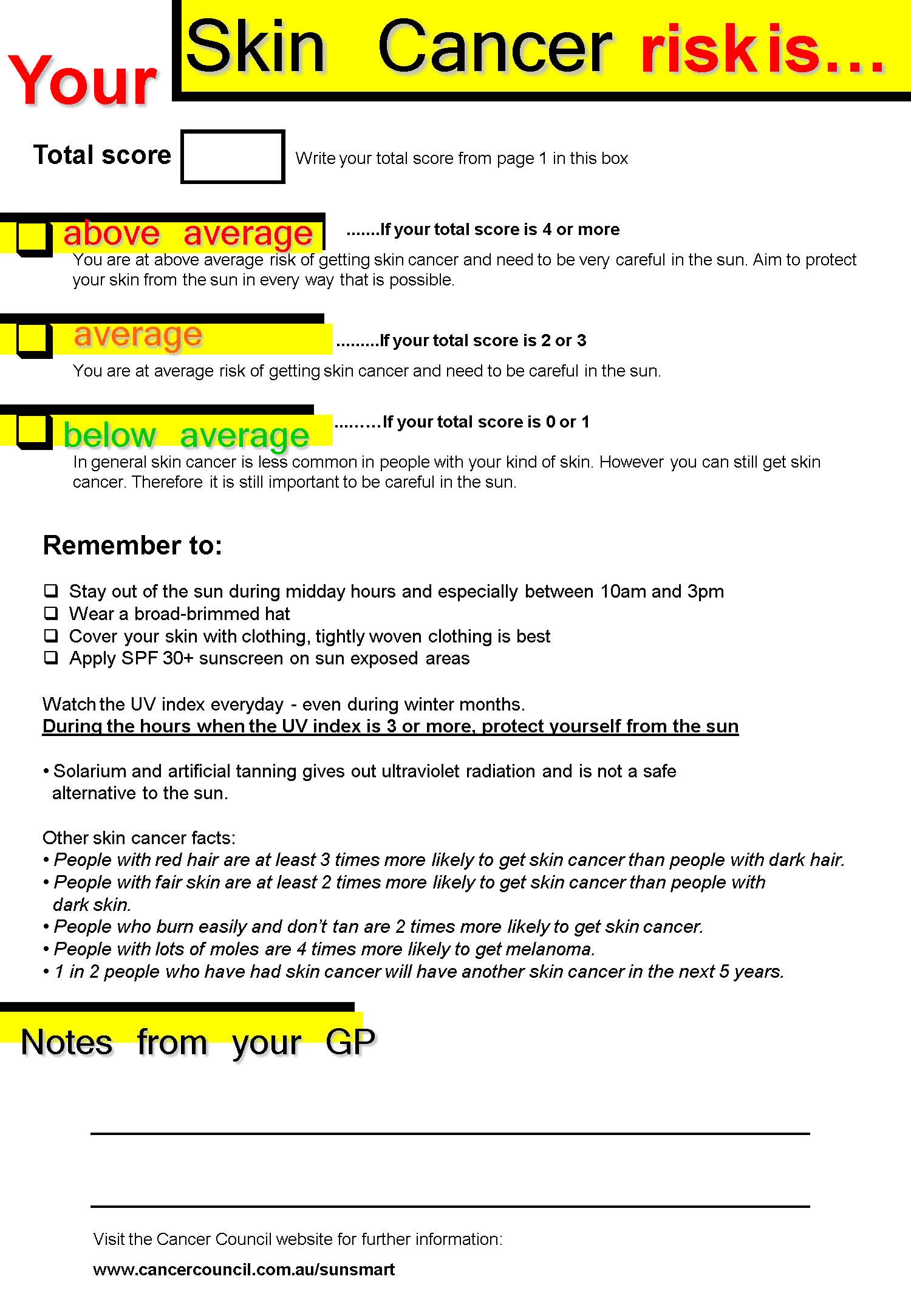

Supplement: Additional file 1 — Skin cancer risk assessment tool used in two Sydney general practices in 2010. [file 1471-2296-15-137-S1.doc]
