# Supplementary material for: Feasibility of a GP delivered skin cancer prevention intervention in Australia
Source: BMC Fam Pract. 2014 Jul 28;15:137. doi: 10.1186/1471-2296-15-137 (PMC4128422; doi:10.1186/1471-2296-15-137)
Supplement: Additional file 2 — Sun habits survey. [file 1471-2296-15-137-S2.doc]

Additional file 2: Sun habits survey

| 1. | People with olive skin will not get skin cancer. | True |
| --- | --- | --- |
|  |  | False |
|  |  | Not sure |
| 2. | You can only get sunburns during sunny days. | True |
|  |  | False |
|  |  | Not sure |
| 3. | Solarium tanning increases the chance of getting skin cancer. | True |
|  |  | False |
|  |  | Not sure |
| 4. | Sunscreen works immediately when applied. | True |
|  |  | False |
|  |  | Not sure |
| 5. | A base suntan reduces the changes of skin cancer. | True |
|  |  | False |
|  |  | Not sure |
| 6. | Loosely woven clothing is the best type of material for sun protection when in the sun. | True |
|  |  | False |
|  |  | Not sure |
| 7. | In the last year, have you received some information on skin cancer? | No |
|  |  | Yes, if yes where did you get it? |
| 8. | If I were to ever get skin cancer, I would need surgery. | Strongly agree |
|  |  | Agree |
|  |  | Neutral |
|  |  | Disagree |
|  |  | Strongly disagree |
| 9. | If I were to ever get skin cancer, I would be in a lot of pain. | Strongly agree |
|  |  | Agree |
|  |  | Neutral |
|  |  | Disagree |
|  |  | Strongly disagree |
| 10. | Over-exposure to the sun will speed up the ageing process. | Strongly agree |
|  |  | Agree |
|  |  | Neutral |
|  |  | Disagree |
|  |  | Strongly disagree |
| 11. | In your opinion, what are your chances of getting skin cancer in the next 10 years? | Less than 20% |
|  |  | 20-40% |
|  |  | 41-60% |
|  |  | 61-80% |
|  |  | 81-100% |
| 12. | In your opinion, what are your chances of getting skin cancer in your whole lifetime? | Less than 20% |
|  |  | 20-40% |
|  |  | 41-60% |
|  |  | 61-80% |
|  |  | 81-100% |
| 13. | If I follow sun protection advice I would reduce my chance of developing skin cancer. | Strongly agree |
|  |  | Agree |
|  |  | Neutral |
|  |  | Disagree |
|  |  | Strongly disagree |
| 14. | I find it difficult to remember to put on sunscreen before I go outside. | Strongly agree |
|  |  | Agree |
|  |  | Neutral |
|  |  | Disagree |
|  |  | Strongly disagree |
| 15. | Wearing a hat is inconvenient. | Strongly agree |
|  |  | Agree |
|  |  | Neutral |
|  |  | Disagree |
|  |  | Strongly disagree |

| 16. | I find it difficult to protect myself from the sun. | Strongly agree |
| --- | --- | --- |
|  |  | Agree |
|  |  | Neutral |
|  |  | Disagree |
|  |  | Strongly disagree |
| 17. | A tan is attractive. | Strongly agree |
|  |  | Agree |
|  |  | Neutral |
|  |  | Disagree |
|  |  | Strongly disagree |
| 18. | It feels nice to be in the sun. | Strongly agree |
|  |  | Agree |
|  |  | Neutral |
|  |  | Disagree |
|  |  | Strongly disagree |
| 19. | Most people regularly wear sunscreen for sun protection. | Strongly agree |
|  |  | Agree |
|  |  | Neutral |
|  |  | Disagree |
|  |  | Strongly disagree |
| 20. | Most people try to stay in the shade for sun protection. | Strongly agree |
|  |  | Agree |
|  |  | Neutral |
|  |  | Disagree |
|  |  | Strongly disagree |
| 21. | How often do you wear sunscreen of SPF 30 or more while you are in the sun? | Never |
|  |  | Sometimes |
|  |  | Half the time |
|  |  | Often |
|  |  | Always |
| 22. | How often do you wear a wide-brimmed hat or a cap with a flap while you are in the sun? | Never |
|  |  | Sometimes |
|  |  | Half the time |
|  |  | Often |
|  |  | Always |
| 23. | How often do you wear a long-sleeve shirt while you are in the sun? | Never |
|  |  | Sometimes |
|  |  | Half the time |
|  |  | Often |
|  |  | Always |
| 24. | How often do you wear sunglasses while you are in the sun? | Never |
|  |  | Sometimes |
|  |  | Half the time |
|  |  | Often |
|  |  | Always |
| 25. | How often do you limit your time in the sun during mid-day hours? | Never |
|  |  | Sometimes |
|  |  | Half the time |
|  |  | Often |
|  |  | Always |
| 26. | How many hours would you usually spend outdoors on a weekday and weekend? | Hours per weekday |
|  |  | Hours per weekend day |
